# Safety of Nonsteroidal Anti‐Inflammatory Drug Use in Traumatic Brain Injury

**DOI:** 10.1002/phar.70199

**Published:** 2026-09-11

**Authors:** Jennifer Beavers, Mary Katherine Cella Shultz, Leanne Atchison, Cara Lwin, Dandan Liu, Silky Chotai, Andrew J. Medvecz, Robel Beyene, Michael C. Smith, Bradley M. Dennis, Susan Hamblin

**Affiliations:** ^1^ Department of Pharmaceutical Services Vanderbilt University Medical Center Nashville Tennessee USA; ^2^ Medical Affairs, Cytokinetics San Francisco California USA; ^3^ Department of Biostatistics Vanderbilt University Medical Center Nashville Tennessee USA; ^4^ Department of Neurological Surgery Cleveland Clinic Foundation Cleveland Ohio USA; ^5^ Division of Acute Care Surgery Vanderbilt University Medical Center Nashville Tennessee USA; ^6^ Department of Pharmacy Practice Lipscomb University College of Pharmacy Nashville Tennessee USA

**Keywords:** intracranial hemorrhage, nonsteroidal anti‐inflammatory drug, trauma, traumatic brain injury

## Abstract

**Background:**

Use of nonsteroidal anti‐inflammatory drugs (NSAIDs) after a traumatic brain injury (TBI) is controversial due to potential worsening of an intracranial hemorrhage (ICH). This study evaluated if NSAID use within 14 days of TBI was associated with a clinically significant progression of intracranial bleed (PIB).

**Methods:**

A retrospective, propensity score‐weighted study was conducted in adult patients with a TBI admitted to an American College of Surgeons Certified Level I Trauma Center. The NSAID group included patients who received at least one dose of an NSAID within 14 days of injury while admitted. The control group included patients who did not receive NSAIDs. The primary end point was incidence of a clinically significant PIB (decrease in the Glasgow Coma Score (GCS) > 2 and an increased or new ICH). Secondary outcomes included need for additional neurosurgical intervention and PIB on imaging regardless of GCS change.

**Results:**

978 patients were included: 284 in the NSAID group and 694 in the control group. Most patients had an Abbreviated Injury Score head > 2 (88%) and a subdural hematoma (57%). Fifty‐two percent of patients had a mixed bleed. A clinically significant PIB occurred in 0.35% in the NSAID group and 1.2% in the control group (Hazards Ratio 0.1, 95% Confidence Interval 0.01–0.82, *p* = 0.032). There was no difference in additional neurosurgical operations or PIB regardless of GCS change. In the NSAID group, there was no significant difference in time from admission to NSAID administration between patients who experienced a PIB and those who did not have a PIB (1.9 days vs. 4.7 days, respectively, *p* = 0.06).

**Conclusion:**

In this single‐center, retrospective, propensity score‐weighted study, NSAIDs were not associated with an increased risk of clinically significant PIB in patients with acute TBI when administered within 14 days of injury. NSAIDs appear safe in the acute phase post‐TBI. Prospective studies are needed to validate our results.

## 1 Introduction

Nonsteroidal anti‐inflammatory drugs (NSAIDs) are a crucial component of multimodal analgesia regimens in polytrauma [1]. In addition to the opioid‐sparing benefits of these medications, NSAIDs are an effective treatment option for headaches after traumatic brain injury (TBI) [2, 3, 4]. However, use of NSAIDs after TBI is controversial due to their antiplatelet activity via inhibition of platelet aggregation which may increase bleeding [5, 6].

NSAIDs have historically been avoided in the presence of TBI out of concern for the potential expansion or new development of intracranial hemorrhage (ICH) regardless of their potential benefits as part of a multimodal analgesia regimen [1]. There is a paucity of evidence evaluating the safety of NSAIDs in the acute TBI population; however, available literature suggests that it may be safe to initiate NSAIDs postinjury [7, 8, 9, 10]. Although assessing irreversible antiplatelet agents instead of NSAIDs, one retrospective study demonstrated a low rate of progression of intracranial bleed (PIB) (< 1%) in patients with an acute TBI started on aspirin or clopidogrel (median time from admission to initiation of 4 days) [9]. This further suggests the potential safety of NSAIDs, which have reversible antiplatelet effects, in the acute TBI phase. At our academic medical center, NSAID use in TBI varies, and our current discharge instructions advise patients to avoid NSAIDs for 14 days after injury.

The primary objective of this study was to evaluate whether the use of NSAIDs within 14 days of initial injury in patients with TBI leads to a clinically significant PIB compared to patients who did not receive NSAIDs. We hypothesized that there would be no difference in PIB rates between the NSAID and no NSAID groups.

## 2 Methods

### 2.1 Study Design

We performed a single‐center, retrospective, propensity score‐weighted cohort study for patients with an acute TBI diagnosis admitted to an American College of Surgeons verified Level 1 Trauma Center between January 1, 2018 and August 31, 2021. This study was approved by the Institutional Review Board. Included patients were 18 years or older with an acute ICH seen on a head computed tomography (CT) scan. Patients taking antiplatelet medications or therapeutic anticoagulation prior to admission, died or were deemed nonsurvivable prior to the analysis period, or initiated on antiplatelet medication(s) or therapeutic anticoagulation within 14 days of admission were excluded. Patients were stratified into two cohorts: patients who had inpatient NSAID administration within 14 days of admission (NSAID group) and patients who did not (control group).

### 2.2 Standard of Care

During the study period, the neurosurgery service was consulted for all patients admitted with a TBI. Repeat head CTs were not routinely obtained on all patients. A repeat head CT within 6–24 h could be considered in patients with high‐risk features on their initial CT scan (i.e., subdural hematoma (SDH), epidural hematoma (EDH), intraventricular hemorrhage (IVH), or intraparenchymal hemorrhage (IPH)). It was standard practice to obtain repeat imaging if the patient had any decline in neurologic exam (i.e., unexplained Glasgow Coma Scale (GCS) decline, new focal neurologic sign or seizure, abnormal pupillary exam, or new delirium or agitation). Neurologic exams were performed hourly for patients admitted to the intensive care unit (ICU) and every 2–4 h for those admitted to the stepdown unit. The need for a neurosurgical procedure was at the discretion of the neurosurgery attending. For the NSAID group, the specific agent and formulation administered were at the discretion of the provider and restricted by hospital formulary.

As for concomitant antiplatelet and anticoagulant medications, all patients were initiated on chemoprophylaxis for venous thromboembolism (VTE) with enoxaparin 72 h after admission. Patients with an intracranial pressure monitor (ICPm), external ventricular drain (EVD), thoracic epidural (TEC), or renal dysfunction with a creatinine clearance < 30 mL/min received subcutaneous heparin for VTE chemoprophylaxis. Although patients who received antiplatelet medications and therapeutic anticoagulation within 14 days of admission were excluded, given that the analysis period could extend beyond 14 days, concomitant use of antiplatelet medications and therapeutic anticoagulation after 14 days was collected.

### 2.3 Outcomes

The primary outcome of this study was the incidence of a clinically significant PIB, defined by a decrease in Glasgow Coma Scale (GCS) > 2 and a repeat head CT scan demonstrating expansion of a known hemorrhagic lesion or development of a new ICH using the ABC/2 method [11]. Secondary outcomes included PIB regardless of GCS change, need for repeat neurosurgical intervention, and readmission for PIB. Neurosurgical intervention was defined as burr holes, craniotomy, craniectomy, or new ICPm or EVD placement.

### 2.4 Statistical Analysis

**FIGURE 1:**
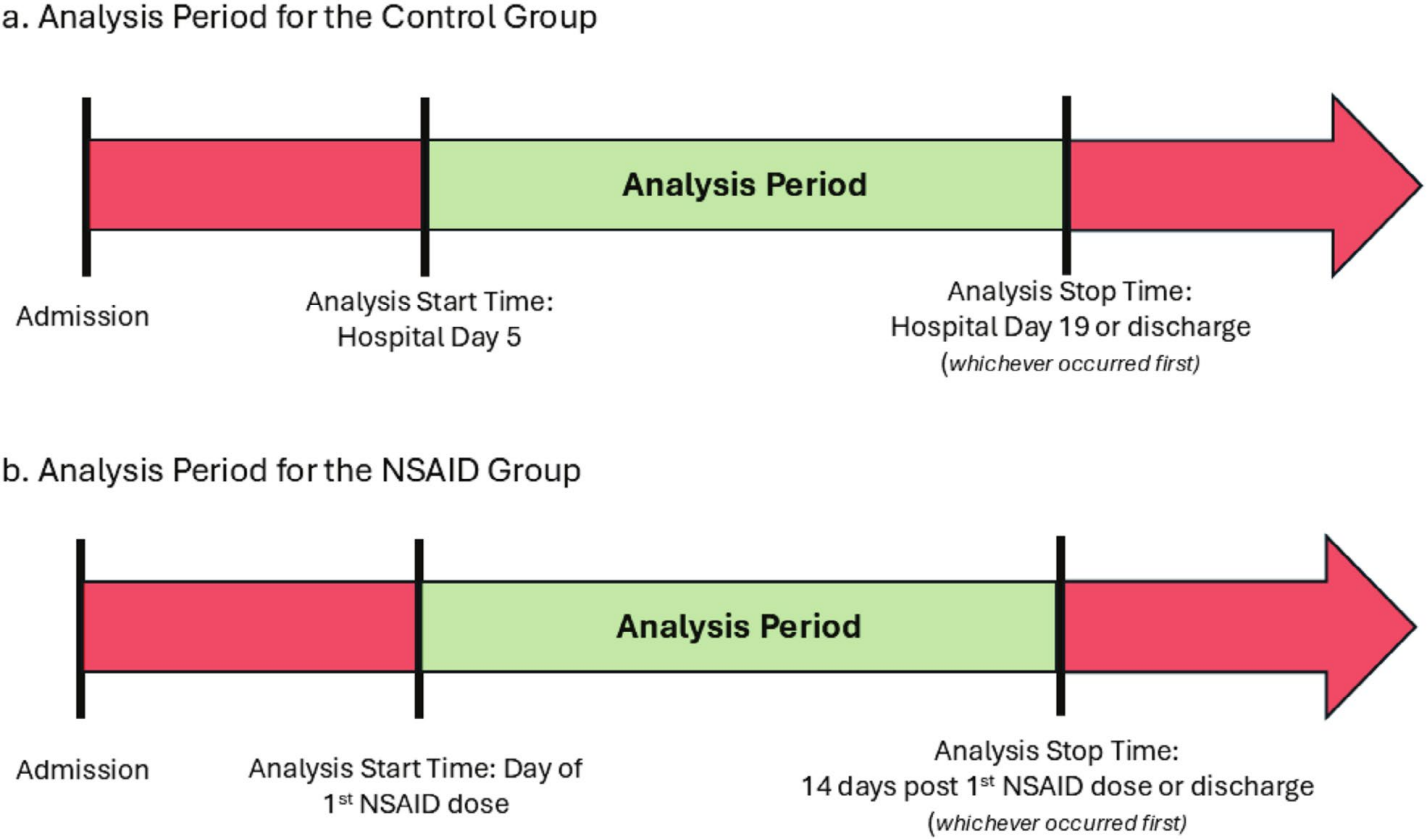
(A) For the control group, the primary and secondary outcomes were assessed from hospital Day 5–19 or hospital discharge, whichever occurred first. (B) For the NSAID group, the primary and secondary outcomes were assessed from the first day of NSAID administration until 14 days after the index NSAID administration or hospital discharge, whichever occurred first. NSAID, nonsteroidal anti‐inflammatory drug.

For the NSAID group, the primary and secondary outcomes were assessed from the first day of NSAID administration until 14 days after the index NSAID administration or hospital discharge, whichever occurred first. For the control group, the primary and secondary outcomes were assessed from hospital Day 5 until Day 19 or hospital discharge, whichever occurred first (Figure 1). Hospital Day 5 was selected because it was the median time to initiation of an NSAID in the NSAID group. This was done to account for the expected progression of an ICH and reduce immortal time bias between the cohorts.

The patient was considered to have a readmission for PIB (verified by worsening or new ICH on imaging) if the readmission occurred 5–19 days from the initial injury for the control group or within 14 days of the first NSAID administration in the NSAID group. For patients readmitted with a PIB in either study group, NSAID usage post discharge was assessed based on documentation in the History and Physical notes by the Emergency Medicine, Trauma, and Neurosurgery services and in the Pharmacy Medication History note if available.

Mean imputation for missing variables was used in the propensity score‐weighted and cox‐proportional hazards model. For the propensity score‐weighted model, logistic regression on NSAID use was conducted to estimate the probability of a patient receiving an NSAID. Average treatment effect (ATE) weights were created based on the propensity scores. Variables associated with NSAID administration and included in the propensity score were age, injury type, injury severity score (ISS), abbreviated injury score‐head (AIS head), AIS thoracic, AIS extremity, TBI severity, bleed type including mixed versus single bleed, neurosurgical intervention required on admission, average daily morphine milligram equivalents (MME), acute kidney injury, acute pain service consult, butalbital/acetaminophen/caffeine administration for TBI headache, and spine surgery. We performed a Cox proportional hazards survival model to determine the NSAID exposure effect on time to the primary and secondary outcomes, where time zero is defined as the day of NSAID exposure for the exposure group and Day 5 for the control group. Death is treated as a competing risk event.

To determine potential risk factors for PIB in the NSAID group alone, characteristics for those who experienced a PIB were compared to those who did not. For this analysis, Wilcoxon (Mann–Whitney) and Fisher's exact test were used for continuous and categorical variables, respectively. A *p*‐value of less than 0.05 was considered statistically significant. Analyses were performed using R version 4.6.0 (R Foundation for Statistical Computing, Vienna, Austria).

## 3 Results

### 3.1 Patient Population

**FIGURE 2:**
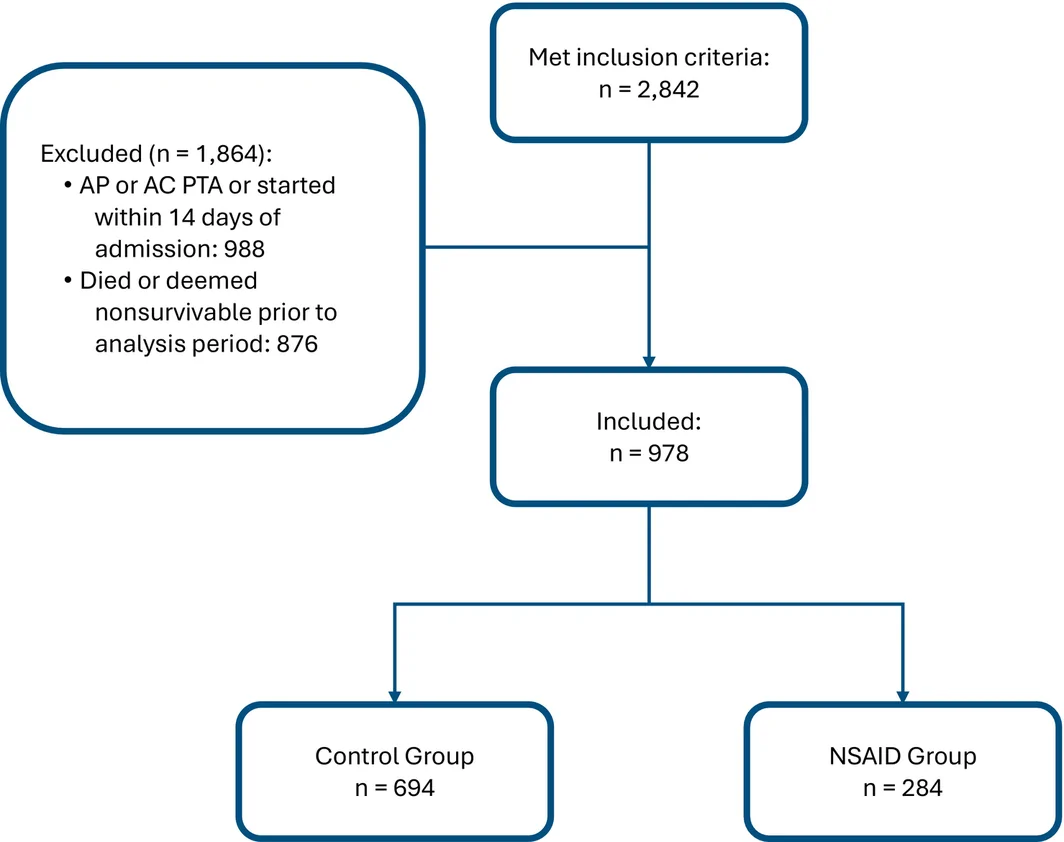
Inclusion and exclusion flow diagram. AP, antiplatelet; AC, anticoagulation; PTA, prior to admission.

In total, 2842 patients met inclusion criteria. 1864 patients were excluded primarily due to use of antiplatelet or anticoagulation medications prior to admission. The final analysis included 978 patients, with 694 patients in the control cohort and 284 patients in the NSAID cohort (Figure 2).

Baseline characteristics are described in Table 1. Most patients were male and white with a median age of 48 (interquartile range (IQR) 31–65) years old. Largely, patients had a mild TBI secondary to blunt injuries with a median head AIS of 4 (IQR 3–4) and ISS of 22 (IQR 17–29); however, there were more patients with a severe TBI in the NSAID group (43% vs. 33%) compared with the control group. SDH and mixed bleeds accounted for half of the bleeding patterns overall. The majority of patients in both groups had a repeat head CT (61%) whereas a relatively limited number of patients initially required a craniotomy, craniectomy, or burr holes (11%). Overall, 32 patients (3.3%) received concomitant antiplatelet medications or therapeutic anticoagulation at some point during the analysis period. Both cohorts had a similar number of patients experience a PIB prior to the analysis period (19% control group vs. 17% NSAID group).

**TABLE 1:** Baseline characteristics.

	Control (*N* = 694)	NSAID group (*N* = 284)	Overall (*N* = 978)
Gender			
Female	206 (30)	80 (28)	286 (29)
Age, years	52 (32, 69)	41 (29, 54)	48 (31, 65)
Race			
Unknown	21 (3)	6 (2)	27 (3)
Asian	4 (1)	4 (1)	8 (1)
Black or African American	98 (14)	30 (11)	128 (13)
White	571 (82)	244 (86)	815 (83)
TBI Severity			
Mild	406 (59)	143 (50.4)	549 (56)
Moderate	57 (8)	18 (6.3)	75 (8)
Severe	231 (33)	123 (43.3)	354 (36)
Hospital LOS, days	8 (5.0, 17.6)	10.5 (6.8, 18.5)	9 (5.5–17.8)
Head AIS			
5	154 (22.2)	52 (18)	206 (21.1)
4	215 (31)	84 (30)	299 (30.6)
3	248 (35.7)	105 (37)	353 (36.1)
2	76 (11)	42 (15)	118 (12.1)
1	1 (0.1)	0 (0)	1 (0.1)
Injury Severity Score	22 (16, 29)	24 (17, 30)	22 (17, 29)
Bleed category			
SDH	390 (56.2)	167 (59)	557 (57)
EDH	28 (4)	3 (1)	31 (3)
Both	24 (3.5)	11 (4)	35 (4)
Other bleed type	252 (36.3)	103 (36)	355 (36)
Mixed bleed	357 (51)	148 (52)	505 (52)
Initial EVD or ICP monitor	101 (15)	50 (18)	151 (15)
Initial Craniotomy, craniectomy, or burr holes	81 (12)	22 (8)	103 (11)
Patients with repeat CTH	427 (62)	174 (61)	601 (61)
Concomitant antiplatelet medication	14 (2)	11 (4)	25 (3)
Concomitant therapeutic anticoagulation	3 (0.43)	4 (1)	7 (0.7)
PIB Prior to Analysis	131 (19)	49 (17)	180 (18.4)
Time to PIB (h)	7.3 (3.4–49)	4.5 (3–13.4)	6.1 (3.1–42.5)

*Note:* Values are represented as number (%) and median (IQR). Mean imputation for missing variables was used in the propensity score‐weighted and cox‐proportional hazards model. One patient in the NSAID cohort did not have an ISS or Head AIS.

Abbreviations: AIS, abbreviated injury scale; CTH, computed tomography of the head; EDH, epidural hematoma; EVD, external ventricular drain; ICP, intracranial pressure; LOS, length of stay; NSAID, nonsteroidal anti‐inflammatory drug; PIB, progression of intracranial bleed; SDH, subdural hematoma; TBI, traumatic brain injury.

For the NSAID group, the median time from admission to the first NSAID dose was 4.6 days (2.4–7). The median number of NSAID doses was 5 (2–12) with ibuprofen and intravenous ketorolac being the most commonly used NSAIDs. The characteristics of NSAID administration are displayed in Table 2.

**TABLE 2:** NSAID administration.

Outcome	NSAID group (*n* = 284)
Time from admission to first administration (days)	4.6 (2.4–7)
Total number of doses	5 (2–12)
NSAID type	
Ibuprofen	166 (58.4)
Ketorolac IV	136 (48)
Ketorolac PO	15 (5.3)
Other^a^	12 (4.2)
Multiple NSAID types	40 (14)
Frequency	
Scheduled	207 (73)
As needed	209 (74)
Mixed	42 (15)

Abbreviations: IV, intravenous; NSAID, nonsteroidal anti‐inflammatory drug; PO, by mouth.

^a^ Other NSAIDs include naproxen, celecoxib, and meloxicam. Values are represented as number (%) and median (IQR).

### 3.2 Primary and Secondary Outcomes

In total, nine (0.9%) patients had a clinically significant PIB, with one (0.35%) patient in the NSAID group and eight (1.2%) in the control group (Hazards Ratio (HR) 0.1, 95% Confidence Interval (CI) 0.01–0.82, *p* = 0.032) (Table 3). Of these nine patients, two patients developed a new ICH while the remaining seven patients experienced an increase in their ICH size.

**TABLE 3:** Primary and secondary outcomes.

	Control (*N* = 694)	NSAID group (*N* = 284)	Overall (*N* = 978)	HR (95% CI)	*p*
Primary outcome					
Clinically significant PIB	8 (1.2)	1 (0.35)	9 (0.9)	0.1 (0.01–0.82)	0.032
Secondary outcomes					
New or Worsening ICH (regardless of GCS change)	17 (2.5)	4 (1.4)	21 (2.1)	0.68 (0.13–3.48)	0.64
New ICPm or EVD^a^	2 (0.3)	0 (0)	2 (0.2)	N/A	N/A
New Craniotomy or Burr Holes	2 (0.3)	1 (0.4)	3 (0.3)	0.42 (0.04–4.7)	0.48
In‐hospital mortality	43 (6.2)	28 (9.9)	71 (7.3)	2.15 (1.26–3.66)	0.005
Readmission^a^	7 (1)	0 (0)	7 (0.7)	N/A	N/A

*Note:* Values are represented as numbers (%). Mean imputation for missing variables was used in the propensity score‐weighted and cox‐proportional hazards model. One patient in the NSAID cohort did not have an injury severity score (ISS) or Head‐Abbreviated Injury Score (AIS Head). Due to a limited number of patients having the outcome, covariates of interest were included in the propensity score model rather than in the cox‐proportional hazards model: age, injury type, ISS, AIS Head, AIS thoracic, AIS extremity, TBI severity, bleed type including mixed vs. single bleed, neurosurgical intervention required on admission, average daily morphine milligram equivalents, acute kidney injury and, need for acute pain service consult, butalbital/acetaminophen/caffeine administration for TBI headache, and spine surgery.

Abbreviations: CI, confidence interval; EVD, external ventricular drain; GCS, Glasgow coma scale; HR, hazards ratio; ICH, intracranial hemorrhage; ICPm, intracranial pressure monitor; N/A, not applicable; NSAID, nonsteroidal anti‐inflammatory drug; PIB, progression of intracranial bleed.

^a^ Due to zero events occurring in the NSAID cohort, the Cox model exhibited complete separation and parameter estimates were unstable.

The secondary outcomes are listed in Table 3. New or worsening ICH regardless of GCS change occurred in 1.4% versus 2.5% in the NSAID and control group, respectively (HR 0.68, 95% CI 0.13–3.48, *p* = 0.64) with the overall median time to worsening or new ICH from admission of 6.9 days (IQR 5.7–12.7). Two patients in the control group and zero patients in the NSAID group required repeat EVDs or ICPm placement. In addition, 0.4% in the NSAID group required a new craniotomy or burr holes due to a worsening ICH compared to 0.3% of patients in the control group (HR 0.42, 95% CI 0.04–4.7, *p* = 0.48).

There were no readmissions for PIB in the NSAID group, and seven patients in the control group were readmitted. Of note, one patient in the control group had taken aspirin and caffeine 845/65 mg oral powder packet 2 days prior to readmission. A second patient in the control group may have been taking aspirin 325 mg daily prior to readmission; however, this was unclear based on the documentation in the electronic medical record. For the patients who received concomitant anticoagulation or antiplatelet therapy during the analysis period, none experienced a PIB.

Overall, 71 (7.3%) patients died, 43 in the control group and 28 in the NSAID group (HR 2.15, 95% CI 1.26–3.66, *p* = 0.005). However, when patients who received their first NSAID dose after transitioning to the palliative care service were excluded from this analysis, there was no difference in in‐hospital mortality (HR 1.37, 95% CI 0.75–2.52, *p* = 0.31) between groups. Two patients in the control group died due to a PIB, one likely from hyponatremia secondary to syndrome of inappropriate antidiuretic hormone secretion (SIADH) leading to increased edema and the second patient due to a new IPH. None of the patients in the NSAID group died due to a PIB.

In the NSAID group, no potential risk factors for PIB were identified, including AIS head, NSAID type, bleed category, or time to first NSAID administration (Table 4).

**TABLE 4:** Potential risk factors for PIB in the NSAID group.

	No PIB (*N* = 280)	PIB (*N* = 4)	*p*
Age, years	41 (29–54)	55.5 (42–67)	0.24
ISS^a^	24 (17–30)	24 (19–28)	0.8
AIS head^a^			
5	51 (18.1)	1 (25)	0.37
4	84 (29.9)	0 (0)	
3	103 (36.7)	3 (75)	
2	42 (15)	0 (0)	
1	0 (0)	0 (0)	
NSAID type			
Ibuprofen	163 (58.2)	3 (75)	0.64
Ketorolac oral or intravenous	144 (51.2)	2 (50)	1
Other^b^	9 (3.2)	0 (0)	1
Multiple NSAIDs	39 (13.9)	1 (25)	0.46
Bleed category			
SDH	163 (58)	4 (100)	0.43
EDH	3 (1.1)	0 (0)	
Both	11 (3.9)	0 (0)	
Other bleed type	103 (36.8)	0 (0)	
Initial GCS	12 (3–15)	14.5 (13.8–15)	0.15
Time from Admission to First NSAID, days	4.7 (2.5–7)	1.9 (1.6–2.6)	0.06
Total NSAID doses	5 (2–12)	9 (6.5–11.5)	0.33

*Note:* All values are represented as number (%) or median (IQR).

Abbreviations: AIS, abbreviated injury score; EDH, epidural hematoma; GCS, Glasgow Coma Scale; ISS, injury severity score; NSAID, nonsteroidal anti‐inflammatory drug; PIB, progression of intracranial bleed; SDH, subdural hematoma.

^a^ One patient in the NSAID cohort did not have an ISS or Head AIS.

^b^ Other NSAIDs include naproxen, celecoxib, and meloxicam.

## 4 Discussion

In this retrospective, propensity score‐weighted cohort study comparing the incidence of a clinically significant PIB in patients who received an NSAID within 14 days of injury versus patients who did not, NSAID use was not associated with an increased risk of developing a clinically significant PIB. To our knowledge, this is the largest study to date analyzing the safety of NSAIDs after a TBI. In addition, our study provides the most data on ketorolac use post‐TBI as 53% of patients received ketorolac intravenously or orally. This data is especially important as ketorolac is often a critical component of multimodal analgesia, yet clinicians may be concerned about the risk of bleeding with this drug in particular.

Other studies have also alluded to the safety of NSAID use post‐TBI. A secondary analysis of the Multi‐modal Analgesic Strategies in Trauma (MAST) trial found no association between administration of an NSAID within 24 h of admission and PIB [10]. In addition, a retrospective study assessing the progression of ICH in blunt trauma patients with a single bleed type who received ibuprofen postinjury found no difference in progression of ICH (ibuprofen group 4.8% vs. control 9.2%; *p* = 0.49) [7].

We did find an increased risk of in‐hospital mortality in the NSAID group (HR 2.15, 95% CI 1.26–3.66, *p* = 0.005). However, several patients had received the initial NSAID dose after transitioning to the palliative care service. When these patients were removed from the analysis, there was no difference in in‐hospital mortality between the two cohorts (HR 1.37, 95% CI 0.75–2.52, *p* = 0.31).

Studies assessing the safety of antiplatelet use, such as aspirin and P2Y12 inhibitors, post TBI can potentially be extrapolated to predict the effects of NSAIDs post TBI. One retrospective study evaluated hemorrhagic complications after starting aspirin or clopidogrel in patients with an acute TBI. The median ISS was 21 with 66% of the population diagnosed with a mild to moderate TBI. The median time to antiplatelet therapy initiation was 4 days postinjury. The study found that new or worsening acute hemorrhages were rare (0.45%) with antiplatelet therapy use [9]. Our similar rate of PIB (0.35%) in a comparable patient population receiving NSAIDs further supports the safety of NSAIDs post TBI.

Studies assessing the effects of preinjury antiplatelet medications on TBI outcomes have been mixed with some showing preinjury antiplatelet medication use to be associated with a higher risk of ICH, poor functional outcomes, and mortality [12, 13, 14]. However, a recent systematic review and meta‐analysis did not demonstrate an increase in ICH progression, mortality, or need for neurosurgical intervention in patients on a single antiplatelet agent [15]. Preinjury NSAID use such as ibuprofen has not been found to be associated with concussion risk, severity, recovery, ICH progression, or need for neurosurgical procedure [8, 16].

Overall, 18.4% of patients developed a PIB prior to the analysis period (Table 1). Interestingly, developing a PIB did not deter NSAID initiation as 17% of patients who had PIB were started on NSAIDs after the initial worsening repeat imaging. None of these patients had a second PIB after receiving NSAIDs. Likewise, in the secondary analysis of the MAST trial, 26% of patients who developed a PIB were still administered an NSAID after the PIB [10]. This same study similarly found no association between the timing of NSAID administration and the development of PIB. This suggests that even after developing a PIB, patients can safely receive NSAIDs.

In our study, most PIB occurred within 24 h from admission with median time to PIB of 6.1 h (IQR 3.1–42.5) from admission (Table 1). Our findings correlate with other studies which identified that ICH progression most commonly occurs in 30% to 51% of patients within 24 h of injury [17, 18, 19]. We chose the analysis period to start at the time of NSAID administration to 14 days after the first NSAID dose for the NSAID group and from hospital Day 5 to Day 19 for the control group, based on the median hospital day an NSAID was initiated. By starting the analysis period for the control group at the median time to NSAID administration, we aimed to prevent capturing a PIB that would have happened regardless of the study group.

Our study does have several limitations to note. This is a retrospective study that relied on accurate documentation of GCS to identify a clinically significant PIB. Second, there was no protocol in place guiding NSAID use including patient selection, drug selection, or dosing in patients with TBIs during the study period; therefore, NSAID prescribing practices varied between providers, which could have caused selection bias towards patients with less severe TBIs receiving more NSAIDs. We controlled for this with a propensity score‐weighted model that estimated the probability of a patient receiving an NSAID. In addition, there were more patients with a severe TBI in the NSAID group, leading us to conclude that selection bias did not significantly affect the cohorts. Third, not all patients had repeat imaging to evaluate for worsening or new ICH as repeat head CTs at our institution are reserved for patients with a decline in neurologic status or high‐risk features on their admission imaging. Therefore, patients in either cohort may have experienced an expansion of a known ICH or development of a new ICH that was not captured. We accounted for this by defining our primary outcome of clinically significant PIB as a decline in neurologic status (i.e., decrease in GCS of > 2) with a progression or new ICH on repeat imaging as this would identify patients who clinically declined due to a worsening TBI versus those who remained clinically stable despite worsening on imaging. Lastly, the specific NSAID agent used was at the discretion of the provider and hospital formulary; thus, conclusions on the safety of NSAIDs not used to in this study (e.g., indomethacin, etodolac, diclofenac, and salsalate) are unable to be made.

## 5 Conclusion

NSAIDs are a crucial component of multimodal analgesia regimens in polytrauma, yet the persistent concern that their antiplatelet effects will worsen bleeding associated with TBI has led to some avoidance of NSAID use in this population. In our study, we found no difference in the incidence of clinically significant PIB in patients with acute TBI who received an NSAID while admitted to the hospital within 14 days of the initial injury. Although prospective, randomized controlled trials are warranted to validate our results, our study suggests NSAIDs can be safely initiated in patients with a TBI within the initial period of hemorrhagic monitoring.

## Author Contributions

**Jennifer Beavers:** conceptualization, investigation, funding acquisition, writing – original draft, methodology, validation, visualization, writing – review and editing, project administration, data curation, supervision, formal analysis, resources. **Mary Katherine Cella Shultz:** conceptualization, investigation, funding acquisition, writing – original draft, methodology, validation, visualization, writing – review and editing, data curation, formal analysis, project administration. **Leanne Atchison:** conceptualization, investigation, methodology, visualization, writing – review and editing. **Cara Lwin:** software, formal analysis, methodology, writing – review and editing. **Dandan Liu:** methodology, software, formal analysis, writing – review and editing. **Silky Chotai:** investigation, writing – review and editing, data curation, validation, methodology. **Andrew J. Medvecz:** writing – review and editing, visualization, formal analysis. **Robel Beyene:** methodology, writing – review and editing, conceptualization, visualization, formal analysis. **Michael C. Smith:** methodology, conceptualization, writing – review and editing, visualization, formal analysis. **Bradley M. Dennis:** writing – review and editing, conceptualization, methodology, visualization, formal analysis. **Susan Hamblin:** funding acquisition, conceptualization, investigation, writing – original draft, methodology, validation, visualization, writing – review and editing, formal analysis, project administration.

## Funding

This study was supported in part by the Vanderbilt CTSA grant UL1TR002243 from NCATS/NIH.

## Disclosure

AI use: No AI tools were used in the drafting of this manuscript, tables, or figures.

## Conflicts of Interest

The authors declare no conflicts of interest.

## Data Availability

The data that support the findings of this study are available from the corresponding author upon reasonable request.
